# Megalin: A Sidekick or Nemesis of the Kidney?

**DOI:** 10.1681/ASN.0000000572

**Published:** 2024-11-15

**Authors:** Kalyani Kulkarni, Tahir Hussain

**Affiliations:** Department of Pharmacological and Pharmaceutical Sciences, College of Pharmacy, University of Houston, Houston, Texas

**Keywords:** albuminuria, kidney, kidney disease, proteinuria, renal proximal tubule cell

## Abstract

Megalin is an endocytic receptor in the proximal tubules that reabsorbs filtered proteins in the kidneys. Recycling of megalin after endocytosis and its expression on the apical plasma membrane of the proximal tubule are critical for its function. The expression of megalin in the kidney undergoes dynamic changes under physiologic and pathophysiologic conditions. Receptors and various effector signaling components regulate megalin expression and, potentially, function. Genetic manipulation and rare mutations in megalin suggest that a lack of or deficiency in megalin expression/function promotes tubular proteinuria and albuminuria. However, the role of megalin in kidney diseases associated with obesity, diabetes, hypertension, and nephrotoxicity remains unclear. To address these questions, animal and human studies have indicated megalin as a protective, injurious, and potentially urinary marker of nephropathy. This article reviews the literature on the regulation of megalin expression and the role of megalin in the pathophysiology of the kidney under experimental and clinical conditions. Moreover, this review articulates the need for studies that can clarify whether megalin can serve as a therapeutic target, in one way or the other, to treat kidney disease.

## Introduction

Protein filtration through the glomerulus, particularly low molecular weight (LMW) proteins, and their reabsorption at the proximal tubules is a normal process of kidney physiology. The reabsorbed proteins are transendocytosed or directed to lysosomal degradation.^1–3^ The reabsorbed proteins, which serve as carrier and binding proteins for vitamins, such as vitamin D, and several hormone molecules, are also reabsorbed and send these vitamins and hormones back into the blood circulation.^4–6^ Albumin,^7^ insulin, vitamin D–binding protein, hemoglobin, retinol-binding protein, *β*2 microglobulin, and antiapoptotic protein survivin^8^ are some of the proteins that are reabsorbed. Thus, this process helps maintain homeostasis of these essential molecules and prevents protein wasting. This reabsorption is mediated by the fast recycling endocytic transporter megalin, which is a member of the LDL receptor family and is highly expressed in the proximal tubules of the kidney.^9,10^ Megalin functions alone and in concert with other proteins, mainly cubilin, which is anchored to the plasma membrane by amnionless^11^ and forms a functional membrane receptor complex cubam.^11,12^ Megalin has broader ligand specificity than cubilin and has an endodomain. In addition to proteins, megalin also binds with numerous toxic ligands, such as gentamicin, aprotinin^13–15^ aminoglycosides, glycated proteins, and myeloma light chain,^3^ and transports them back to the proximal tubules and circulation.

Megalin recycling is a highly controlled process regulated by various adapter proteins such as autosomal recessive hypercholesterolemia, adaptor protein complex-1, Disabled-2, oculocerebrorenal syndrome protein 1, Rab 11,^10^ and kinases such as glycogen synthase kinase 3, protein kinase C (PKC), and protein kinase A.^16^ With the help of these proteins and enzymes, clathrin-coated pits and early and late endosomes determine the fate of megalin, particularly recovering it back to the cell surface, which is required to maintain its normal transport function.^17^ Along with megalin, cubilin is endocytosed and recycled back to the plasma membrane.^9,18,19^ Reduced expression of megalin, such as under excessive protein/albumin absorption, caused by shedding^15,20^ and/or reduced transcription^20^ likely reduces protein reabsorption and promotes proteinuria.^21^

Generally, proteinuria is considered the gold standard clinical marker of kidney injury^22^ and is also a risk factor of tubulointerstitial injury and cardiovascular diseases.^23–25^ Tubular proteinuria, however, may not necessarily contribute to tubulointerstitial injury, especially under glomerular proteinuria and protein hyperfiltration^26–28^; rather, it could protect against proximal tubular stress caused by excessive protein uptake and metabolic load.^26–28^ Although much has been published and reviewed for the role of megalin in proteinuria and kidney disease, this mini review is a direct attempt to analyze and delineate the literature to clarify whether megalin is protective/sidekick or injurious/nemetic to the kidney and under what pathophysiologic conditions.

## Megalin Expression

It is important that megalin cell surface expression is maintained to prevent proteinuria and loss of essential molecules, such as vitamins.^4–6^ However, numerous studies have revealed that renal expression of megalin is higher in certain pathologic conditions, whereas it is lower in others, and thus may have functional implications in tubular proteinuria and tubulointerstitial injury.

### Upregulation

**Figure 1:**
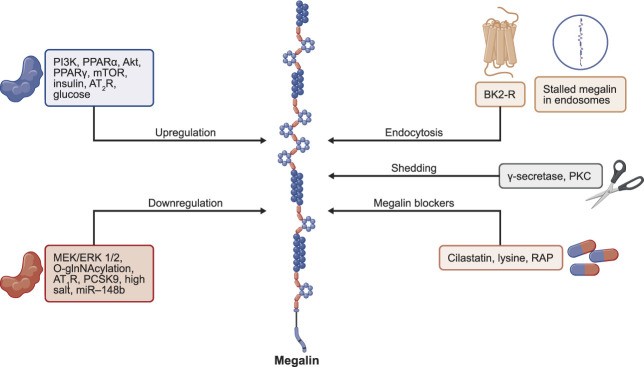
**Receptors, effector molecules, and drugs as regulators of megalin by various mechanisms, namely altering its expression, shedding it from the plasma membrane, endocytosis, and stalling it in the cytoplasm and blocking its binding with ligands in the kidney and *in vitro* proximal tubule epithelial cells.** This scheme is based on the literature cited in this review. AT_1_R, angiotensin II type 1 receptor; AT_2_R, angiotensin II type 2 receptor; BK2-R, bradykinin type 2 receptor; miR-148b, mRNA-148b; mTOR, mammalian target of rapamycin; PCSK9, proprotein convertase subtilisin/kexin type 9; PI3K, phosphoinositide 3-kinase; PKC, protein kinase C; PPAR*α*/*γ*, peroxisome proliferator–activated receptor; RAP, receptor-associated protein.

Most studies on diabetes have shown higher megalin expression in the kidneys. Specifically, streptozotocin-induced type 1 diabetic rats and Zucker fatty rats with type 2 diabetes showed enhanced kidney mRNA expression of megalin.^29,30^ High glucose concentration and oxidative stress, which are associated with diabetes, have been proposed to drive megalin upregulation, potentially through the phosphoinositide 3-kinase (PI3K)/Akt pathway (Figure 1), as tested in human kidney (HK-2) proximal tubule epithelial cells.^29^ Mammalian target of rapamycin (mTOR) is downstream of the insulin/PI3K pathway^31,32^ and protects against cell surface megalin reduction and LMW proteinuria,^33^ as shown by long-term treatment with the mTOR inhibitor rapamycin in mice. In addition, glucose alone and in the presence of insulin upregulated megalin expression in these cells.^30^ However, in epithelial-like pig kidney (LLC-PK1) cells, glucose has been shown to reduce megalin protein and cell surface expression levels.^34^ Megalin studies in HK-2 cells can be questioned in light of the reports that HK-2 cells do not express megalin.^35^ Activation of the transcription factors proxisome proliferator–activated receptor (PPAR)*γ* and PPAR*α* increases megalin protein expression in the mouse kidney.^36^ Insulin has also been reported to increase megalin protein and mRNA expression through the PI3K pathway.^37^ Angiotensin II type 2 receptor (AT_2_R) activation protects megalin protein expression in obese rats fed a high-salt diet.^19^

### Downregulation

Megalin downregulation and reduced cell surface protein and mRNA expression have been reported in salt-fed rats and mice^19,38,39^ and in the proximal tubule epithelial cell line LLC-PK1.^40^ In a mouse study, a 7-day high-salt diet feeding led to a decrease in megalin expression in the kidney.^39^ However, high-salt diet feeding was associated with greater oxidative stress and inflammation, which could have affected megalin expression. Although oxidative stress causes upregulation of megalin expression,^29^ inflammation caused by lipopolysaccharides or TNF-*α* decreases megalin expression, potentially through the Mitogen-activated protein kinase 1 and 2 and extracellular signal–regulated kinase 1 and 2 pathway and transcriptional mechanism.^41^ In obese rats, 2 days of high-salt diet feeding reduced megalin protein and cell surface (Figure 1) expression on the apical plasma membrane potentially because of recycling impairment and lysosomal degradation as megalin was found to be localized with the lysosomal membrane protein lysosomal associated protein-1.^19^ In this study, high-salt diet–induced reduction in megalin expression and lysosomal localization of megalin was prevented by AT_2_R activation and was associated with increased activities of Akt and glycogen synthase kinase 3*β*, which cause megalin phosphorylation and recycling impairment.^19,42^ By contrast, angiotensin II type 1 receptor (AT_1_R) activation reduces megalin protein and mRNA expression through the extracellular signal–regulated kinase 1 and 2 pathway, as shown in opossum kidney cells.^37^ In patients with IgA nephropathy, renal megalin mRNA and mRNA-148b (miR-148b) are downregulated, which is associated with CKD progression.^43,44^ In another study, unilateral ureteral obstructed mice kidneys, which resulted in miR-148b upregulation but a decrease in tubule megalin expression.^45^ This inverse relationship between miR-148 and megalin was confirmed by *in silico* mRNA target prediction analysis. Moreover, the LLC-PK1 cells transfected with miR-148b reduced megalin mRNA and protein levels, suggesting a direct role of miR-148b in megalin downregulation.^45^

Bradykinin receptor-2 stalls megalin in the early endosome (Figure 1) through the PKC pathway, leading to a reduction in megalin cell surface expression and thus a reduced albumin transport function.^40^ Another mechanism that downregulates megalin cell surface expression is O-GlnNAcylation using Akt under high glucose concentrations present in the media of LLC-PK1 kidney epithelial cells^34^ and in spontaneously hypertensive rat kidneys with albuminuria and proteinuria.^46^ Contrary to these studies, reduced O-GlnNAcylation of megalin in response to sodium-glucose cotransporter-2 inhibitors leads to reduced membrane expression and function of transport proteins.^47^ Nevertheless, megalin O-GlnNAcylation reduces its expression on the plasma membrane, likely because of megalin recycling impairment.^47^ Although there is no measurement of megalin expression in the HK-2, urinary levels of megalin were found to be higher in patients with diabetic kidney disease or cisplatin-induced nephrotoxicity.^48^ A study revealed that megalin expression in the kidneys of nephrotic patients due to shedding was associated with higher protein synthesis turnover and megalin mRNA.^20^ Megalin shedding in urine, which occurs by sequential actions of metalloproteinases, PKC, and *γ*-secretase (Figure 1), may release endodomain in the cytoplasm, which in turn translocates to the nucleus,^49,50^ regulates gene expression, and suppresses megalin expression.^49^ Specifically, opossum kidney cells stably transfected with membrane-bound megalin *C*-terminal fragments or soluble megalin intracellular domains showed reduced protein and mRNA expression of megalin and the apical Na-transporter Na,H-exchanger-3 (NHE3).^51^ Because NHE3 is inversely related to megalin recycling efficiency, it is likely that reduced NHE3 expression increases megalin recycling and function. By contrast, a study in mice expressing megalin endodomains did not show a role in regulating gene expression, suggesting a differential role of endodomains in *ex vivo* versus *in vivo* studies.^52^ Proprotein convertase subtilisin/kexin type 9 (PCSK9), a ubiquitously expressed cholesterol-regulating protein that downregulates LDL receptors,^53,54^ is filtered by the glomerulus and interacts with megalin, leading to reduced cell surface and protein expression (Figure 1), thereby causing proteinuria.^55^ The PCSK9 knockout and knock-in studies suggested an inverse relationship between PCSK9 and megalin expression levels,^55^ which was further supported by the observation that in nephrotic syndrome, elevated plasma PCSK9 levels were associated with reduced megalin expression (Figure 1) with the potential for higher proteinuria and nephrotic syndrome.^55^

Overall, it seems that numerous effector molecules and receptors are involved in regulating megalin expression under various pathologic conditions. Consistent with the injurious versus protective role of AT_1_R and AT_2_R in kidney function, AT_1_R decreased megalin protein expression and AT_2_R protected megalin protein and cell surface expression and function. Activation of the transcription factor PPAR*γ*, which is linked to numerous hormone receptors including AT_2_R,^56,57^ reduces insulin resistance and improves glycemia in type 2 diabetes by regulating insulin receptor signaling–related genes and upregulates megalin expression in the kidney.^16,36,56,57^ Although findings from *ex vivo* studies are helpful in understanding the mechanisms of megalin regulation, more studies in animal and HK-2 biopsies with various pathologic conditions are needed to understand the factors and cellular mechanisms responsible for megalin regulation, recycling impairment, and shedding into urine.

## Kidney Disease: Is Megalin Protective or Injurious?

Proteinuria/albuminuria as end points, their genesis, and the mechanisms of kidney disease are complex, making it difficult to pinpoint the precise role of megalin in these processes. However, numerous studies in humans and animal models, including genetic models, have attempted to correlate and/or establish a cause-and-effect relationship between megalin expression and proteinuria/albuminuria and kidney injury under various pathologic conditions (Table 1). In this article, we summarize human and animal studies that have suggested that megalin could be protective and/or injurious to the kidneys.

**Table 1:** Protective or injurious role of megalin in humans with genetic disorders and various animal models

Model	Megalin Protective/Injurious
**Humans**	
LRP2 variant: DB/FOAR with LMW proteinuria and kidney injury^58^	Protective
**Animals**	
Kidney-specific megalin knockout mice: (no comorbidity): increased GFR, albuminuria, and kidney injury^58^	Protective
Pharmacologic regulators of megalin (without comorbidity): PPAR*α* and PPAR*γ*,^36^ AT_1_R blocker,^75^ AT_2_R agonist,^19^ TGF1*β via* SMAD2/3^76^, mTOR^33^—increased megalin expression, prevent proteinuria, and tubulointerstitial injury	Protective
Pharmacologic regulators of megalin (podocin-knockout nephrotic mice): PCSK9 inhibitor alirocumab—increased megalin expression and reduced albuminuria^55^	Protective
Kidney-specific megalin knockout (obesity, immunotoxin, cystenotic kidney): proteinuria, decreased nephrotic syndrome, FSGS and reduced accumulation of toxic products in proximal tubules, and reduced tubular injury^80,84,89^	Injurious
Pharmacologic blockers of megalin: cilastatin and lysine—prevent nephrotoxicity by vancomycin, colistin, cisplatin, gentamycin, and cystine^85,88^	Injurious

AT_1_R, angiotensin II type 1 receptor; AT_2_R, angiotensin II type 2 receptor; DB/FOAR, Donnai–Barrow and facio-oculo-acoustico-renal; LMW, low molecular weight; LRP2, low-density lipoprotein receptor–related protein 2; mTOR, mammalian target of rapamycin; PPAR*α*/*γ*, peroxisome proliferator–activated receptor.

### Human Studies

#### Protective

While pathogenic variants of low-density lipoprotein receptor–related protein 2 (*LRP2*) encoding megalin are known to cause a rare autosomal recessive syndrome known as Donnai–Barrow and facio-oculo-acoustico-renal, which includes kidney dysfunction in terms of LMW proteinuria and increased kidney injury markers (kidney injury molecule-1 and NAGase),^58^ a recent study of human biopsy samples revealed a protective role of megalin in the progression of CKD.^58^ In patients with Lowe syndrome, which is caused by a mutation in the *OCRL1* gene, which encodes phosphatidylinositol 5-phosphatase,^59^ there are reports of LMW proteinuria^59^ associated with decreased urinary excretion of megalin^60,61^ and accumulation of megalin in the proximal tubule cells,^62^ suggesting megalin recycling impairment and reduced endocytic function. Reduced renal expression of megalin has also been reported in nephrotic patients with proteinuria,^20^ suggesting an inverse relationship. Imerslund–Gräsbeck syndrome is characterized by mutations in *CUBN*, intestinal vitamin B12 malabsorption, and proteinuria. In this case, albuminuria seems to be benign because the patients have normal kidney function and do not require proteinuria-lowering treatment.^63^ All these studies indicate a protective role of megalin and cubilin in proteinuria. However, the patients in these studies did not seem to have comorbidities, such as diabetes, causing excessive protein/albumin filtration.

Bardoxolone methyl is a potent activator of Nrf2^64–66^ and produces antioxidant, anti-inflammatory response and improves GFR, BUN, and other kidney function markers in patients with CKD, but these improvements were associated with albuminuria, strong inflammation, and disease progression.^67^ Primate studies indicated that bardoxolone treatment for 1 year and Nrf2 activation cause megalin downregulation, which contributes, in part, to albuminuria.^68^ However, megalin downregulation does not seem to adversely affect the kidney structure.^68^

#### Correlation between Urinary Megalin and Nephropathy

Few studies performed in the Japanese diabetic population suggest a positive correlation between urinary megalin excretion and diabetic nephropathy and the extent of nephropathy, as determined by various kidney injury markers, such as GFR and proteinuria.^69^ Furthermore, megalin in the urine is a marker of glomerular abnormalities in various glomerular diseases and IgA nephropathy.^70,71^ Similarly, urinary levels of cubilin also correlate with proteinuria and loss of vitamin-binding protein and vitamin D3 in patients with preeclampsia. However, another study suggested no correlation between urinary megalin levels and eGFR in diabetic and nondiabetic patients.^72^ Overall, most studies suggest that greater the loss of megalin in urine, higher the decline in eGFR and nephropathy. It is to be debated whether increased megalin excretion in urine is the result of proximal tubule cell damage caused by an excessive filtered metabolic load and thus serves only as a marker and not a cause of progressive kidney disease.

### Animal Studies

#### Protective

Mice with conditional megalin knockout specific to the kidney had significantly lower GFR and higher plasma creatinine associated with kidney injury markers, such as kidney injury molecule-1 and NAGase.^58^ Other studies in kidney-specific megalin knockout mice have revealed higher proteinuria and albuminuria.^73,74^ However, these studies did not report kidney injury. In a mouse model of subAKI, albumin overload leads to tubulointerstitial injury, which is associated with reduced megalin expression, and the AT_1_R blocker losartan restores megalin expression and prevents tubulointerstitial injury.^75^ PPAR*α* and PPAR*γ* agonists also restore megalin expression in proximal tubules and prevent proteinuria and tubulointerstitial injury in bovine serum albumin–injected rats.^36^ Reduced megalin expression has been reported in fibrosis-associated pathologies, such as diabetic nephropathy and hepatic fibrosis.^76^ Streptozotocin-induced diabetic rats exhibited albuminuria, type 4 collagen deposition, and decreased megalin expression in the kidneys. These effects were reversed by injecting soluble fragments of TGF1*β* receptor type 2 through small mothers against decapentaplegic 2 and 3 transcription factors.^76^ Although these studies showed a strong association between reduced megalin expression and kidney injury/fibrosis, it is unclear whether megalin restoration preceded injury/fibrosis or *vice versa*. Likewise, it is also unclear whether the pharmacologic agents ARB, PPAR*γ* agonist, and TGF*β*1 fragment restored megalin expression first and the reversal of the injury followed it, or it is the other way around. Because mTOR inhibition is implicated in reducing megalin expression and proteinuria,^33^ other studies revealed that mTORC1 may not affect the levels of megalin and cubilin, but it may affect the endocytic machinery resulting in Fanconi-like syndrome of glucosuria, phosphaturia, aminoaciduria, LMW proteinuria, and albuminuria,^77^ which is consistent with megalin knockout studies.^73,74^ Proximal tubule cell–specific deletion of megalin (using *Lrp2* floxed [*Lrp2 f/f*] mice and Cre transgenic mice expressing a tamoxifen-inducible Cre recombinase under the control of Ndrg1 promoter) showed tubulointerstitial nephritis in mice fed a Western diet,^78^ suggesting a protective effect of megalin. Megalin knockout (KO) male mice fed with Western diet (high fat and refined sugar) showed a compromised kidney function and a significant increase in kidney injury as compared with control mice, implicating a protective role of megalin.^79^ Overall, these studies suggest that megalin is potentially protective against tubular interstitial injury and fibrosis. Because some studies are simply correlative, a more direct approach may be needed to determine the role of megalin in the initiation and/or progression of kidney diseases.

#### Injurious

Contrary to the potential protective effects of megalin, several studies implicate megalin in proximal tubule cells to be injurious. In a study with glomerular injury in which megalin knockout mosaic mice (60% of the proximal cells lacking megalin) were crossbred with NEP25 (transgenic mice expressing human CD25 in podocytes)^80^ and injected with immunotoxin, nephrotic syndrome and FSGS developed, which were associated with massive nonselective proteinuria and tubular injury.^80^ However, megalin-expressing cells accumulated more albumin, Ig light chains, IgA, IgG, and injury markers (heme oxygenase-1 and monocyte chemoattractant protein-1) than non–megalin-expressing cells,^80^ suggesting a role for megalin in protein accumulation and cellular injury. Another megalin-deficient mouse study showed a reduced accumulation of proteins and profibrotic markers in megalin-deficient proximal tubule cells, but megalin deficiency did not a prevent tubulointerstitial injury in this model of GN.^81^ A study using a rat model of albumin overload and an *in vitro* study suggested that proteinuria by megalin-mediated lysosomal dysfunction and inflammasome activation leads to inflammation, contributing to the progression of CKD.^82^ In models of early diabetes in the normoalbuminuric stage, oxidative stress causes megalin upregulation, which may be responsible for excessive albumin uptake and diabetic nephropathy.^29^ In another model of long-term diabetes, megalin upregulation is regarded as a molecular representative of tubular injury and the progression of diabetic nephropathy,^83^ although its role in nephropathy has not been postulated. One of the studies implicating megalin directly in kidney injury comes from high-fat–induced kidney disease and kidney-specific mosaic megalin knockout mice.^84^ Megalin KO mice exhibit attenuated autolysosomal dysfunction, hypertrophy, lipid peroxidation, senescent markers of proximal tubule epithelial cells, peritubular capillary rarefaction, interstitial fibrosis, glomerular hypertrophy, and mesangial expansion in response to a high-fat diet. Megalin KO mice had obesity, dyslipidemia, and hyperglycemia similar to those in high-fat diet–fed controls, ruling out the role of these metabolic parameters.^84^ However, another study showed that megalin causes metabolic dysfunction as shown by the findings that megalin KO mice fed with Western-style diet had an improved glucose tolerance and reduced body weight as compared with the controls.^79^ Another study has reported that glucose and insulin enhanced megalin expression.^30^ Whether such an increase in megalin expression contributes to tubular damage is yet to be elucidated.

Blocking megalin by chemicals and supplements has been shown to be renoprotective against toxic drugs. For example, vancomycin, colistin, cisplatin, and gentamicin are taken up by megalin and thereby induce nephrotoxicity. Cilastatin is a megalin blocker (Figure 1) that effectively suppresses the nephrotoxicity induced by these drugs.^85^ One of the molecular mechanisms associated with reduced nephrotoxicity includes receptor-associated protein binding with megalin with high affinity^86^ and reducing the uptake of nephrotoxic drugs.^87^ Megalin function inhibition in adult cystenotic mice supplemented with lysine reduces renal cystine accumulation and protects cystenotic kidneys.^88^ Another study used a conditional excision of floxed megalin/*Lrp2* alleles in proximal tubular cells in a cystenotic mouse model using a *Cre-LoxP* strategy with *Wnt4-CRE* and suggested an injurious role of megalin in this mouse model.^89^

## Conclusion

Megalin expression in the kidney is affected by changes in physiologic and pathophysiologic conditions, such as diabetes and a high-salt diet. Various molecular entities, including receptors and effector molecules, alter megalin expression and potentially affect endocytic function. Specifically, pharmacologic manipulation of molecular entities by drugs, such as PPAR agonists, TGF*β*1, mTOR inhibitor, AT_1_R antagonist, AT_2_R agonist, and nephrotoxic drug blockers, regulates megalin expression and function and affords renoprotection. Considering the dynamic nature of megalin expression and function under pathophysiologic conditions, it is clear that megalin is not a sidekick, rather it is an active player and serves as a nemesis of the kidney, if its expression and function is not targeted to maintain the proximal tubule functional homeostasis. Thus, megalin is clearly an emerging viable target when considering drug regimens for treating kidney diseases. However, more work needs to be conducted in human and animal kidneys to clarify pathologic nuances of cellular and molecular regulatory mechanisms of megalin expression and function.
